# Relationship between Car-Sickness Susceptibility and Postural Activity: Could the Re-Weighting Strategy between Signals from Different Body Sensors Be an Underlying Factor?

**DOI:** 10.3390/s24041046

**Published:** 2024-02-06

**Authors:** Merrick Dida, Michel Guerraz, Pierre-Alain Barraud, Corinne Cian

**Affiliations:** 1 Université Grenoble Alpes, Université Savoie Mont Blanc, CNRS LPNC UMR 5105, 73000 Grenoble, Francecorinnecian@gmail.com (C.C.); 2 Université Grenoble Alpes, CNRS, CHU Grenoble-Alpes, Grenoble INP, TIMC-IMAG, 38041 Grenoble, France; pierre-alain.barraud@univ-grenoble-alpes.fr; 3 Institut de Recherche Biomédicale des Armées, 91223 Brétigny sur Orge, France

**Keywords:** motion sickness, body sensors, multisensory integration, postural control

## Abstract

Postural control characteristics have been proposed as a predictor of Motion Sickness (MS). However, postural adaptation to sensory environment changes may also be critical for MS susceptibility. In order to address this issue, a postural paradigm was used where accurate orientation information from body sensors could be lost and restored, allowing us to infer sensory re-weighting dynamics from postural oscillation spectra in relation to car-sickness susceptibility. Seventy-one participants were standing on a platform (eyes closed) alternating from static phases (proprioceptive and vestibular sensors providing reliable orientation cues) to sway referenced to the ankle-angle phases (proprioceptive sensors providing unreliable orientation cues). The power spectrum density (PSD) on a 10 s sliding window was computed from the antero-posterior displacement of the center of pressure. Energy ratios (ERs) between the high (0.7–1.3 Hz) and low (0.1–0.7 Hz) frequency bands of these PSDs were computed on key time windows. Results showed no difference between MS and non-MS participants following loss of relevant ankle proprioception. However, the reintroduction of reliable ankle signals led, for the non-MS participants, to an increase of the ER originating from a previously up-weighted vestibular information during the sway-referenced situation. This suggests inter-individual differences in re-weighting dynamics in relation to car-sickness susceptibility.

## 1. Introduction

A variety of predictors of Motion Sicknesses (MS) have been examined over the years, among them the characteristics of postural control. Several postural parameters measured in the absence of motion have been correlated to MS’s self-reported history of inertial motion. Differences in sway path length [1,2], power spectral density (PSD) profile [1] and temporal dynamics of the body sway (detrended fluctuation, [3]) were observed between individuals who were likely to suffer MS and those who were never sick. Furthermore, the way individuals adapt and de-adapt to postural perturbations (i.e., continuous oscillations applied to a platform) correlates with MS history susceptibility [4]. Although this may be speculative, it is suggested that individuals without motion sickness adjust their balance control in the perturbation phase and, therefore, take longer to return to their initial postural control. Moreover, subjects with a high degree of habituation to sea sickness show changes in their postural control strategy during exposure to motion [5]. This adaptation in terms of postural control reflects the ability to select the appropriate sensory information from different body sensors [6]. Flexibility in postural adjustments, which may be associated with the “sensory re-weighting” mechanisms [7], would be a necessary condition.

In accordance with the re-weighting principles, loss of balance is prevented in situations of degraded sensory information by decreasing the weight of the unreliable senses and giving more weight to the reliable ones (e.g., [8,9,10,11,12,13,14,15,16,17,18]). Thus, previous research on motion sickness susceptibility suggests that adaptation to changes in the environment may be critical for MS individuals. They may show deficits in the re-weighting of multimodal sensory information during periods of transition in the sensory context, where sensory weighting is not appropriate for postural control. For instance, a transient decrease in postural stability has been reported after both loss and recovery of accurate sensory information due to slow adjustments of sensory weights resulting in an under- or over-generation of corrective torque [16]. As an example, during an eyes-closed stance on a platform that moves to and from a fixed condition to a sway-referenced condition (the platform was made unstable with servo-driven ankle sway referencing with the goal of keeping the ankle angle constant), the relative contributions of the vestibular and proprioceptive sensors to postural control are dynamically regulated. Indeed, to maintain balance during a sway referenced condition, the source of sensory information must rapidly switch from ankle proprioception, which no longer provides relevant cues about the body orientation relative to the earth’s vertical, to vestibular sensors, whose weight is consequently increased [16]. Back to a fixed platform condition, the ankle proprioceptive sensors again provide a measure of the body angle in relation to the earth’s vertical motion. However, as the vestibular weight is higher at this point than during the initial fixed platform phase, the added weights of proprioceptive and vestibular information result in too much corrective torque until optimal sensory re-weighting. For this purpose, the ratio between low- and high-amplitude body sway was computed and its variations as a function of the sensory environment were used as an index of sensory re-weighting [16,19,20]. Accordingly, peaks in specific body sway frequencies can be predicted if sensory re-weighting is inappropriate [16,19]. Using such an approach, we can infer the dynamics of sensory re-weighting from postural oscillation spectra in relation to MS.

We hypothesized that MS individuals have suboptimal abilities to re-weight multimodal sensory information during postural control. In situations where appropriate postural regulation to changing environmental conditions is required, these deficits can lead either to a complete failure of adaptation or to the presence of a transient period of inappropriate sensory weighting. In this context, it is, therefore, essential to understand how susceptibility to MS is related to the temporal aspects of postural regulation, particularly during transient periods of adaptation to changing environmental conditions. To this end, we computed the ratio between body sway at low- and high-frequencies as an index of sensory re-weighting [13,16,19,20] around the onset and offset periods of sway referenced situations. Although motion sickness in road transport is common, it is rarely studied in comparison to, for example, sea sickness. In the present experiment, we focused on the car-sickness susceptibility history of passengers, taking factors such as route, external view and travel activity, that can trigger MS symptoms, into account [21].

## 2. Materials and Methods

### 2.1. Participants

Seventy-one participants (38 women and 33 men) took part in this experiment. They were mainly students of Grenoble Alpes University and were between 18 and 30 years old. Their mean age, height and weight were 20.93 years (SD = 3.13 years), 167.9 cm (SD = 7.97 cm), and 61.48 kg (SD = 8.72 kg), respectively. None of them reported a history of neurological or musculoskeletal disorders that might affect their ability to maintain balance. During the experiment, seven participants (two women and five men) stepped off the side of the force plate or lost their balance. The data from these participants were therefore not considered further, giving a total of 64 participants. All the participants provided their written informed consent before the experiment began. The study was approved by the local independent ethics committee of the University Grenoble-Alpes (CER Grenoble Alpes-Avis-2019-11-05-5). Participants received 10 euros or a 0.75-course credit for their participation, which lasted around 45 min in total.

### 2.2. Experimental Setup

**Figure 1:**
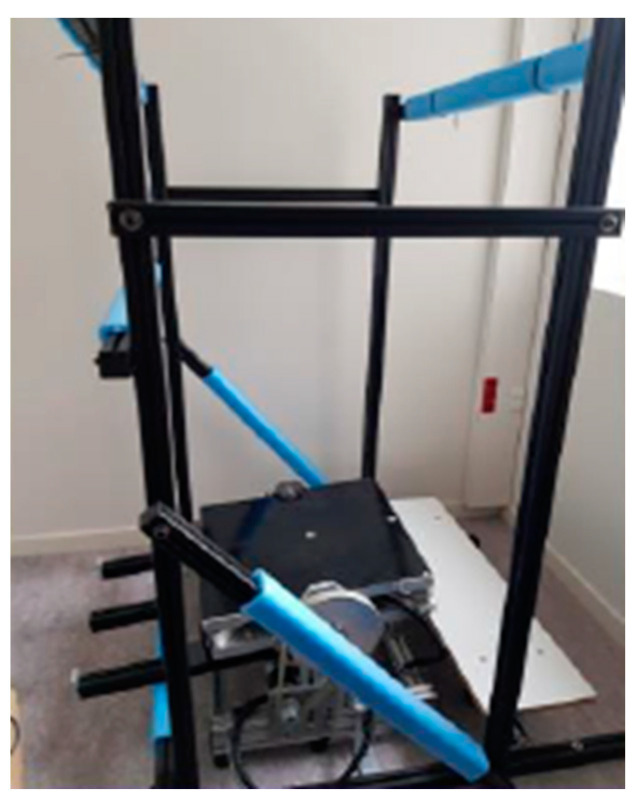
The force platform used in the experimental setup, surrounded by its foam-clad aluminum frame. This platform could be tilted in the AP axis or held stationary horizontally.

The force-platform was custom made from two parallel steel plates (50 × 50 × 3 cm). The upper plate lays on four strain gauges (AG50C3SH10ef SCAIME) mounted near the corners of the lower plate, 40 cm apart. The antero-posterior (AP) and medio-lateral (ML) axes are defined as parallel to the horizontal edges of the platform. This platform could be tilted in the AP axis or held stationary horizontally ([4], Figure 1). The motion of the platform could also be sway referenced. In this condition, the participant’s ankle angle was captured in real time using a Polaris Spectra motion capture device (NDI, Northern Digital Inc.), two passive rigid markers and a pointer probe. The first marker (NDI Rigid Body tool N° 8700449) was placed on the side of the platform to capture its position in real time. A second marker (NDI Rigid Body tool N° 8700339) was positioned on the head of the participant’s left fibula using a knee pad to capture its position in real time. A pointer-type tool (NDI pointer probe N° 8700340) was used to define a virtual third point located on the malleolus. This defined the length of the participant’s fibular segment (from the lateral malleolus to the fibular head and lateral) used to measure any angular displacement around the ankle (malleola). As the platform was coated with anti-slip rubber and the participants were not allowed to move their feet during the experiment, their malleolus remained in the same location relative to the platform, so there was no need for a permanent marker. The position of this segment relative to the plane of the platform (acquired at a frequency of 60 Hz) was then used to calculate the average angle of the participant’s ankle during the first minute (static) of each session. This angle was sent, at a refresh rate of 30 Hz, to the computer controlling the plate to modify the tilt angle of the plate in real time. This experimental setup did not require synchronization between the Polaris acquisition frequency and that of the platform. Indeed, the platform was the slave of the Polaris, the latter collecting the angle of the participant’s ankle and the position of the platform to compute the new position of the platform and transfer this new position to the platform. The platform’s sampling frequency (100 Hz) was higher than that of the Polaris so that it could respond as quickly as possible. The accuracy of the platform tilt angle is less than 0.005 degrees. In this way, the angle of the participant’s ankle was kept approximately constant during the second minute. The time required to process the ankle angle from the Polaris Spectra data and to compute and send a corrective angle to the platform was less than 40 ms. Then, the platform started to adjust its angle in less than 30 ms and reached its goal in less than 30 ms. The total time for the system to perform the sway reference was less than 100 ms. The mean difference between the angle needed at a time t and the real platform angle was less than 0.00116 degrees with a standard deviation of 0.15 degrees. The platform also had a force capture system underneath the plate, consisting of four scales in each of its corners. This allows the recording of the center of pressure (CoP) variations in the horizontal plane. The average accuracy of CoP estimation for a 70 kg load applied to the center of the platform was less than 0.1 mm. These posturographic data were recorded at 100 Hz. In order to prevent falls, the platform was surrounded by a foam-clad aluminum frame to which participants were loosely secured by means of a climbing harness. This device did not interfere with the subject’s free movements.

The participants’ susceptibility to car sickness was assessed using a questionnaire covering five different car situations as a passenger: on winding roads (mountain), on straight roads, when seated in the front passenger seat, when seated in the back of the car and when reading in the car. For each of these five situations, participants had to indicate if they were: never (0), rarely (1), sometimes (2), or often (3) sick. The level of self-reported car sickness of the participants was then constructed as the sum of these five scores. The carsickness score varies between 0 for a participant who would never experience MS in any of these five situations and 15 for a participant who would often be sick in all five situations.

### 2.3. Procedure

After completing the questionnaire, participants had to stand barefoot on the plate, feet placed at shoulder distance, arms at the side of the body and eyes closed. The axis of rotation of the platform was, therefore, collinear with the articulation of their ankles. The position of their feet was marked on the platform to ensure that it remained constant between each trial. Participants were asked to stand still, avoiding bending their knees or hips or moving their arms. They were subjected to three trials of three minutes, each consisting of one minute in the static condition (baseline), one minute of sway reference and finally one minute in the static condition again (return). These three trials were separated into 3-min breaks during which participants were free to get off the platform if they wished. During the sway-reference phase, the maximum achievable angle was limited to +/−5° for safety reasons. Following this phase, the plate returned progressively and linearly (within two seconds) to the horizontal. Finally, at the end of the experiment, they were asked to fill out the misery scale [22], relating to sensations of general discomfort, tiredness, headache, dizziness, stomach discomfort (without nausea), heat and finally, nausea. None of them experienced any of these sensations.

### 2.4. Data Analysis

**Figure 2:**
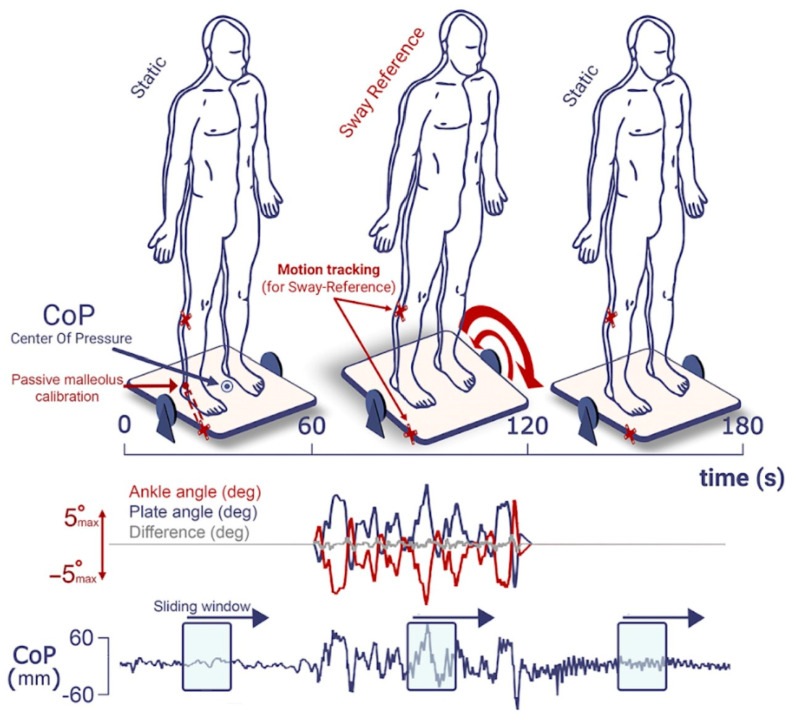
Experimental design and posturographic measurements. The three phases of a given trial (static baseline, sway reference, static return) are represented in the upper panel. The sway reference is performed by the computation of the ankle angle from the two motion tracking markers (in red) positioned on the participant’s fibular head and the platform and the virtual third point pre-calibrated on the malleolus with a pointer-type probe. The time course of this angle, of the plate angle and the difference between the two over one trial, are represented in the red, blue and grey traces, respectively. Below are shown, for each phase, an example of an antero-posterior COP trace with sliding time windows from which power spectral densities (PSDs) and energy ratios (ERs) are computed.

Signal Processing was performed using R (version 4.0.4). For each trial, we computed the short-term power spectrum density (PSD) of the antero-posterior component of the CoP over a sliding time window of 10 s. The PSDs were computed for windows placed at every 10 ms, which corresponds to the sampling frequency of the posturography signal. The psdcore function of the PSD package [23] was used to perform a multitaper estimation of the PSD for each 10 s window. The number of tapers for each frequency was set to 5, which gives a good trade-off between frequency resolution and spectral uncertainty. The PSD is represented by 500 data points between 0.1 Hz and the Nyquist frequency of 50 Hz. For further analysis, we limited the frequency range between 0.1 and 5 Hz (as commonly used in postural studies using PSDs [24]). This interval allows a substantial portion of the CoP energy to be considered while avoiding high-frequency artifacts appearing in the signal around 7 Hz, caused by the mechanical properties of the platform. Each PSD represents the spectrum at the time instant corresponding to the center of the window, so that, for instance, the window spanning from 0 to 10 s yields the PSD for = 5 s. Each PSD is represented by a 50-dimensional vector, whose components are the frequencies of the spectral analysis (from 0.1 to 5 Hz, in steps of 0.1 Hz) and the amplitudes (in dB) of the PSD for the frequencies. Thus, each three-minute trial was represented by a sequence of 17,000 of these vectors (Figure 2).

In order to quantify the distribution of spectral energy, we computed four energy ratios (ER) in time regions of interest. Energy ratios are computed as the ratios of the sum of energy in the high-frequency band (0.7 to 1.3 Hz) to the low-frequency bands (0.1 to 0.7 Hz) over a given 10 s time window. The limits used here are related to specific frequency bands; the lower one reflects undisturbed stance, while the higher one reflects sway activity in response to postural disturbances [16]. The four-time windows were situated at the end of the initial static phase (ER1), the onset of the sway reference phase (ER2), the end of the sway reference phase (ER3) and the onset of the return phase (ER4). The four energy ratios (from ER1 to ER4) were calculated for each trial. Then, these energy ratios were averaged across trials. There was no correlation between the ability to perform the experiment (assessed by the absence of foot movements, knee bends or falls) and car-sickness susceptibility.

### 2.5. Statistical Analysis

According to our hypothesis, the transient periods of adaptation to new sensory environments could be at the core of interindividual variability in MS susceptibility. Thus, we focused our correlational analyses on the relationship between car-sickness susceptibility and the spectral changes due to the onset of the sway reference (loss of relevant ankle proprioceptive information, i.e., the difference between ER2 and ER1) and the return to a static situation (reintroduction of relevant ankle proprioceptive information, i.e., the difference between ER4 and ER3). To complement and further understand the relationships between energy ratios and car sickness, a mixed model analysis of variance was performed with MS sensibility (high versus low MS) as a between-subject factor and the four ER time windows (ER1 to ER4) as a within-subject factor. Low MS participants were selected as below the first quartile with q1 = 1.75 (*n* = 16, 8 men, mean age = 19.94 years (SD = 2.29 years), and high MS participants as above the third quartile with q3 = 10 (n = 11, 1 man, mean age, 20.91 years (SD = 3.36 years) following the method of Hyndman and Fan [25] implemented in R. Statistical analyses were performed using Statistica software (v13.3, 1984–2017, TIBCO-Software INC, StatSoft Europe, Hamburg, Germany), with the significance level set at *p* < 0.05. Post-hoc analyses were performed when necessary (Newman-Keuls test).

## 3. Results

### 3.1. Relation between Car-Sickness Susceptibility and Energy Ratios in Phases of Transition

**Figure 3:**
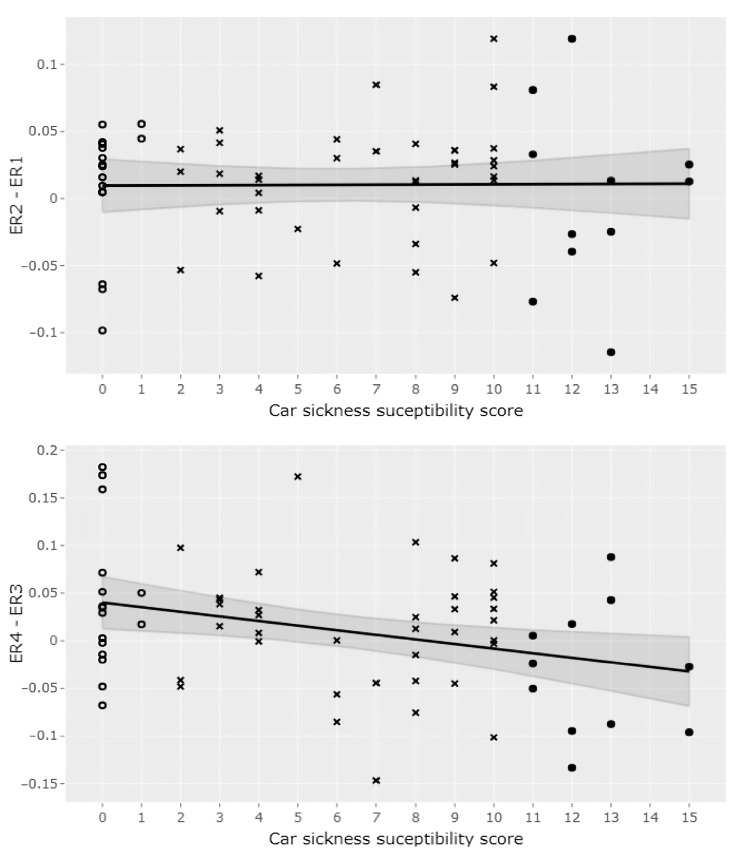
Correlation between ER2-ER1 and ER4-ER3 difference and car-sickness susceptibility score. These two scatter plots represent the relation between the car-sickness susceptibility score and the spectral changes at the loss of the proprioceptive information (ER2-ER1, **top panel**) and at the reintroduction of proprioceptive information (ER4-ER3, **bottom panel**). Hollow circles, plain circles and crosses represent participants in the low car-sickness group (score < Q1 = 1.75), participants in the high car-sickness group (score > Q3 = 10), and participants in the medium car-sickness group (Q1 ≤ score ≥ Q3) respectively. A linear model with a 95% confidence interval was fitted on the data. There is a significant negative correlation (r = −0.26, *p* < 0.05) between ER4-ER3 and the car-sickness score (**bottom panel**), but not between ER2-ER1 and car sickness score (r = −0.04, *p* = 0.74; **top panel**).

While energy ratios followed a normal distribution (Shapiro-Wilk test, *p* > 0.05), it was not the case for the car-sickness scores (Shapiro-Wilk test, *p* < 0.001). Therefore, the Spearman coefficient correlation test was used. The results revealed no correlation (r = −0.04, *p* = 0.74) between car-sickness susceptibility and the difference in energy ratio between the end of the first static phase and the beginning of the sway-reference phase (ER2-ER1). However, a negative correlation was found (r = −0.26, *p* < 0.05) between the end of the sway-referenced phase and the return to a stationary situation (ER4-ER3; Figure 3). Therefore, the fewer participants that were car sick, the higher their ER4 tended to be compared to their ER3.

### 3.2. Postural Differences between Subjects with High and Low Sensitivity

**Figure 4:**
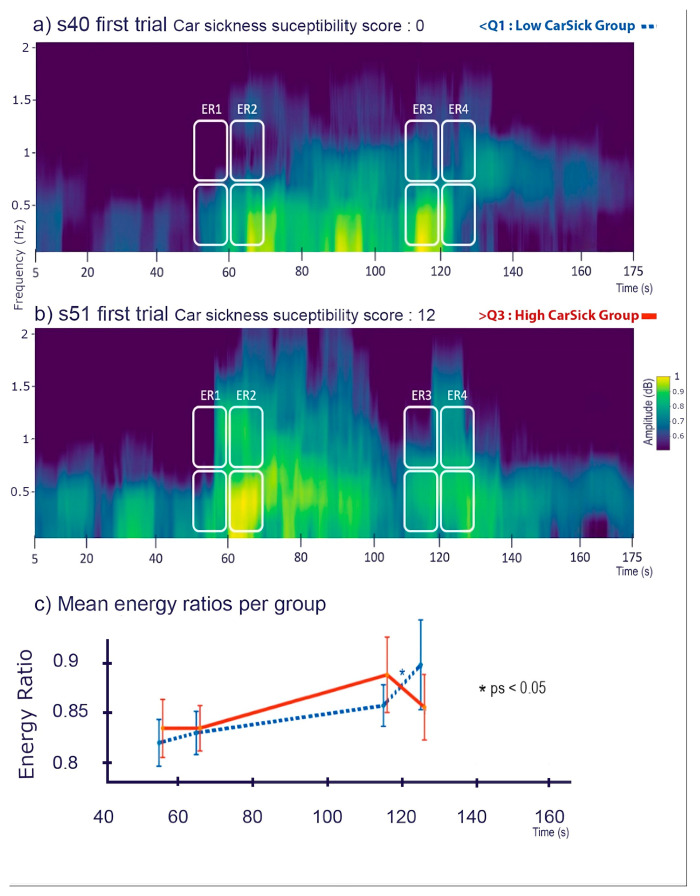
Example spectrograms and mean energy ratios variation between groups: The top (**a**) and middle (**b**) panels, respectively, show examples of spectrograms (frequency x time x energy amplitude) of the first trial of a low car-sick participant (car-sickness score = 0, low car-sickness group) and of a high car-sick participant (car-sickness score = 12, high car-sickness group). Time-frequency regions corresponding to the bands of computation of each energy ratio (ERs) are indicated as white rounded squares. The bottom panel (**c**) displays the evolution of the mean energy ratios per group (low car-sickness group in dotted blue and high car-sickness group in plain red) on the same time axis as prior spectrograms with 95% error bars.

To better describe the relationship between car-sickness and energy ratios, the following analysis focuses on the postural behavior (from ER1 to ER4) of participants with high and low levels of car sickness, defined on the basis of the first and last quartiles of car-sickness susceptibility. The ANOVA showed no main effect of groups (F(1,25) = 0.01 *p* = 0.908). There was a significant main effect of ER over time (F3,75) = 6.93, *p* < 0.001) with no difference between ER1 and ER2 but a significant increase between these first two ratios and the subsequent ER3 and ER4 (*ps* < 0.05), which did not differ from each other (*p* > 0.05). The interaction between ER time windows and groups was marginally significant (F(3,75) = 2.52, *p* = 0.06) (Figure 4). In non-car-sick participants, the energy ratio remained stable from ER1 to ER3 (*ps* > 0.05) but increased when the platform returned to the static situation, with ER4 being significantly higher than ER1 and ER2 (*ps* < 0.05). In contrast, for car-sick participants, the energy ratio remained stable from ER1 to ER4 (*ps* > 0.05).

### 3.3. Postural Differences between Women and Men: A Complementary Analysis

In contrast to the group with low car-sickness susceptibility, which is well balanced by sex, the group with high susceptibility contained only one man. To test whether the postural differences between participants with high and low sensitivity are not confounded with sex differences, a complementary ANOVA was performed with sex (including all participants: 36 women versus 28 men) as a between-subject factor and the four ER time windows (ER1 to ER4) as a within-subject factor. There was no main effect of sex (F(1,62) = 0.46 *p* = 0.502) nor an interaction between sex and ER time windows (F(3,186) = 0.591, *p* = 0.621). In contrast, there was a significant main effect of ER over time (F(3,186) = 10.91, *p* < 0.001) as expected.

## 4. Discussion

The present investigation addressed the potential association between idiosyncratic ways of adapting/de-adapting to postural perturbations and car-sickness susceptibility. Using a sway-referenced situation that impairs ankle proprioception, we observed a negative, albeit weak, relationship between the time–frequency aspects of posture regulation and car-sickness susceptibility in situations where accurate sensory information for orientation was restored, but not in situations where it was lost. By comparing the least car-sick individuals (1st quartile) with the most car-sick individuals (4th quartile), it appeared that the reintroduction of reliable ankle proprioception led to a transient increase in energy ratio only in the non-car-sick participants. This increase can be interpreted as an overproduction of the corrective torque, reflecting a multisensory reorganization.

### 4.1. Sensory Re-Weighting as a Potential Predictor of Car-Sickness Susceptibility

In the context of the inverted pendulum model of standing, (with a single degree of freedom, the ankle angle, and a single motor output, the muscle torque around the ankle), the occurrence of a transient overproduction of the corrective torque (after sway referencing as observed in non-car-sick participants) may be related to sensory re-weighting mechanisms. Each body sensor has an associated weight (w) defining how much they contribute to the postural control. In the absence of vision, as in the present experiment, proprioceptive information is multiplied by a proprioceptive weight (Wp) and vestibular information by a vestibular weight (Wg). The integration of this information is associated with a general weight W (with W = Wp + Wg), yielding a single weighted estimation of the body angle. This estimation feeds a proportional integral derivative neural controller, which can then produce the corrective ankle torque. Since this estimate and the concurrent body angle correction are “imperfect” (due to the inherent noise introduced, whether at the level of the sensors, central processing or motor level), a new weighted estimation will be performed, which will again lead to the generation of a corrective torque, this cycle leading to the production of body sway. The spectrum resulting from body sway is, therefore, directly related to the overall loop gain in the control loop, which results from the sum of the weights (W) given to each sensory signal and the proportional gain of the proportional integral derivative controller. In undisturbed postural conditions, this model predicts that the frequency spectrum of body sway will be essentially flat, that is, without noticeable spectral peaks at low (around 0.1 Hz) or higher (around 1 Hz) frequencies. In order to cope with situations where sensory information is degraded, more weight should be given to the most reliable senses. For example, during sway reference, the proprioceptive inputs from the ankle are inconsistent with the body angle in relation to the earth’s vertical body motion. Thus, Wg must be increased to keep a general weighted information W of around one and to maintain an essentially flat oscillation spectrum. When the sway reference ceases, the proprioceptive information is again accurate. As the vestibular weight is higher at this point than during the initial fixed-platform phase, the added weights of signals provided by the proprioceptive and vestibular sensors lead to W > 1. This would be noticeable in the body sway spectrum by a peak at higher frequencies. This model fits well with the behavior of non-car-sick participants. It can explain the spectral frequency peak observed when the sensory source is suddenly restored.

Participants with a car-sickness history did not differ from non-car-sick participants during the sway reference phase. Both succeeded in restoring normal corrective torque after loss of relevant ankle proprioception, suggesting a process of sensory re-weighting in which the weight allocated to the relevant signals at hand, namely those from the vestibular sensors, increases. However, as Peterka [26] has pointed out, while it is urgent to re-weight the available sensory information when a sensory source of orientation is missing (to avoid instability and reduce the risk of falling) it is much less urgent to re-weight this information once it has been restored since the system is already stable. When the sensory source is suddenly restored, no spectral frequency peaks were observed in car-sick participants, and their ER4 did not increase compared to their ER3, as was the case in non-car-sick participants. Several explanations can be proposed for the lack of high-frequency sway when the stability of the platform has been restored (ER4). According to the sensory re-weighting approach, individuals do not decrease their proprioceptive weight during sway referencing, even though it does not contribute to torque generation. Thus, when sway referencing ended, the proprioceptive component was added immediately to the vestibular component, whose weight was increased during sway reference. This would lead to too much global torque, resulting in higher frequency oscillations. Accordingly, high-frequency sway may be absent from the 10 s post-sway referencing window if the individual was able to reduce their overall sensory weights very quickly. With regard to our main hypothesis, we could suggest that the car-sick participants showed a greater ability to adjust sensory weights in response to changing environmental conditions than the non-car-sick ones. However, this approach does not account for idiosyncratic differences in the proprioceptive and vestibular sensory weights (levels of vestibular vs. proprioceptive contribution to balance). In that context, it can be suggested that the higher the proprioceptive weight, the higher the spectral frequency peak. Thus, the observed differences in the peak size between car-sick and non-car-sick may be related to differences in the proprioceptive and vestibular sensory weights. Accordingly, a lower proprioceptive weight in car-sick participants may explain their lower spectral frequency peak. Finally, the question of whether the proprioceptive weight remains constant during sway referencing is still open. Indeed, several research papers emphasize the importance of dynamic re-weighting to adapt to the environment have shown changes in sensory weights, such as up-weighting relevant sensory cues and down-weighting irrelevant ones [20,27]. Accordingly, during sway referencing, the weight of the irrelevant signals from the proprioceptive sensors may have been reduced. Then, when the sway referencing ended, the total weight of the proprioceptive-generated torque added to the vestibular-generated torque would not be high enough to produce the high-frequency sway. However, in order to detect transient phenomena, data were included that partially overlapped with the time of transitions between the fixed to sway-referenced and vice versa (because of spectra calculation computed on 10 s sliding time windows). This may be seen as a limitation of the method used here, but appears to be the only way of detecting phenomena that are transient in nature [9] that would otherwise be undetectable with a method that clearly separates the analyzed windows.

To maintain balance, car-sick and non-car-sick participants seem to differ in their dynamics of sensory re-weighting. In the different general theories about the etiology of motion sickness, postural control has been shown to reflect susceptibility to MS. On the one hand, the “postural instability” theory of MS has been proposed by Riccio and Stoffregen (1991) [28] based on the relationship between perception and action control. This theory states that MS will be preceded and predicted by instabilities in postural control. On the other hand, motion sickness is thought to be due to conflicts between incoming multimodal sensory information related to body motion, also known as the “sensory conflict” or “neural mismatch” hypothesis [29,30,31]. Conflict occurs when the sensory signals are integrated and found at variance with previously recognized and stored motion paradigms. According to both theories, postural dynamics may reflect susceptibility to MS. However, for the “sensory conflict” hypothesis, postural instability is considered to be the consequence and not the cause of MS [32]. Whether the present experiment highlights differences in the sensory weighting mechanism for postural control in relation to MS susceptibility or not, it does not contradict the “postural instability” theory. Differences in postural control in situations that do not induce MS were also observed in the present experiment. However, the differences concern the organization of sensory systems rather than motor systems for postural stability.

### 4.2. Sex Differences in Susceptibility to Car-Sickness

The group with low levels of car sickness was balanced by sex, whereas the group with high levels of car sickness contained only one man, which is consistent with the greater susceptibility to motion sickness in women compared to men. Indeed, the severity of MS symptoms is greater in women than in men in land and sea transport [21,33,34,35,36]. During road transport, women are also more likely to report sickness than men by a ratio of four to three [21]. However, when motion sickness is visually induced, there is a sex difference in the incidence of motion sickness (assessed using a yes/no forced choice question), but not in its severity (symptoms intensity [37]). One explanation for sex differences in MS susceptibility is the cyclic variations in reproductive hormones during the menstrual cycle [38]. Men and women also differ in body sway [39,40,41,42], which may contribute to sex differences in visually induced motion sickness [43]. However, when all the participants in the present study were included in the analysis (*n* = 64), regardless of their susceptibility to car sickness, the energy ratio between men and women did not differ. Thus, unlike what has been reported for visually induced motion sickness, it seems unlikely that the postural differences observed here between low and high car-sick participants reflect a sex difference.

### 4.3. Relevance of the Car-Sickness Questionnaire

It must be acknowledged that the questionnaire used here lacks experimental validity compared to the Motion Sickness Susceptibility Questionnaire (MSSQ) [33]. However, the MSSQ is defined as the sum of two sub-scores, the MSA (MS susceptibility before 12 years old) and the MSB (MS susceptibility during the last 10 years). Our participants, all engaged in undergraduate studies, were rather young (mean age = 20.93 years, SD = 3.13 years). Thus, in those participants, there would be an overlap between the MSA and MSB. Such an overlap would artificially increase the MSSQ score [44] to a variable extent depending on the age of each participant, with the consequence of inducing a bias in the relationship between motion sickness history and postural control. In addition, we focused on the means of transport (the car) that each participant would have experienced as a passenger in the past five years, rather than covering all types of means of transport as in the MSSQ. Indeed, our participants may not have used some of the different means of transport assessed in the MSSQ in the few years preceding the experiment (or even in their entire lives), which cannot be distinguished from the avoidance of these means of transport due to fear of past motion sickness. For all these reasons, we used a self-made sickness history questionnaire, which is a fairly common method in motion sickness literature (see [45] for a review), with a scoring similar to that of the MSSQ. It would, of course, be an exaggeration to generalize the current results to all means of transport and to the different types of real or apparent movements.

### 4.4. Future Work

Postural responses as an indicator of car sickness have been measured during standing, while individuals are actually seated in cars. However, postural control is not specific to standing, and sitting also involves postural control, although to a lesser extent. Nevertheless, it would be interesting, in future experiments, to take the sitting condition into account in order to study car sickness further in relation to postural control. In addition, vision was not allowed in the present experiment, leading participants to control their balance on the basis of signals from primarily vestibular and proprioceptive sensors. Thus, the re-weighting behaviors highlighted here, which differed between car-sick and non-car-sick individuals, do not necessarily predict what would occur if additional visual signals were provided. Vision is not a primary factor, but rather a secondary etiological factor in the development of motion sickness. Visually impaired people are known to be sensitive, whereas bilateral labyrinthine defective individuals do not suffer from motion sickness [46]. However, as reported by Stoffregen et al. (2013) [3], prior to embarking on a sea voyage, participants who subsequently become sea sick exhibit postural responses to the visible horizon that differ from those of the participants who do not become sick [47]. During road transportation, on the one hand, motion sickness could be significantly reduced by improved forward external vision, although vision alone is not sufficient to completely eliminate motion sickness [21]; on the other hand, reading during travel can induce motion sickness. Therefore, the relationship between postural control and motion sickness may also be related to visual information. It would, therefore, be of particular interest to manipulate visual cues (i.e., vision sway-referenced) in participants with a functional vestibular system to obtain a more comprehensive picture of sensory re-weighting related to motion sickness.

## 5. Conclusions

The present results confirm that the way individuals adapt and de-adapt to postural perturbations correlates with MS history susceptibility. More interestingly, the analysis of energy ratios provided new insights into the underlying mechanisms in play; this analysis showed that non-car-sick individuals adjust their balance control in the perturbation phase and take longer to return to their initial postural control compared to car-sick individuals. These behaviors reveal inter-individual differences in re-weighting dynamics in relation to susceptibility to car sickness. It must be kept in mind that the relationship between car sickness and energy ratios in the current experiment is weak. However, a great number of demographic and physiological factors play a decisive role in the etiology of motion sickness [45]. Therefore, multisensory integration must be seen as an important factor, among others, which contributes to the explanation of interindividual differences in motion sickness.

## Data Availability

All the data and the R scripts used for signal processing are available on OSF at: https://osf.io/f9wg7/?view_only=714b87a295b74c37ba5c9cbc9a35fb03, accessed on 1 January 2024.
